# Sex-specific effects of maternal gestational diabetes mellitus on offspring neurodevelopment: persistent hippocampal neurogenesis deficits in female but not male offspring

**DOI:** 10.1038/s12276-026-01741-z

**Published:** 2026-06-04

**Authors:** Xiafei Wu, Huisheng Ge, Jie Fang, Jie He, Hongbing Xu, Yangyu Zhao, Philip N. Baker, Xinyang Yu, Yubin Ding, Hongbo Qi

**Affiliations:** 1 Department of Obstetrics, The First Affiliated Hospital of Chongqing Medical University, Chongqing, China; 2 Chongqing Key Laboratory of Maternal and Fetal Medicine, Chongqing Medical University, Chongqing, China; 3 Department of Gynecology, Chengdu Women’s and Children’s Central Hospital, School of Medicine, University of Electronic Science and Technology of China, Chengdu, China; 4 Department of Obstetrics and Gynecology, Peking University Third Hospital, Beijing, China; 5 Faculty of Medicine and Health Sciences, University of East Anglia, Norwich, UK; 6 Department of Obstetrics and Gynecology, Women and Children’s Hospital of Chongqing Medical University, Chongqing, China; 7 Department of Pharmacology, Academician Workstation, Changsha Medical University, Changsha, China

**Keywords:** Developmental neurogenesis, Diabetes

## Abstract

Gestational diabetes mellitus (GDM) represents a prevalent pregnancy complication with long-term health implications for offspring. While metabolic outcomes have been extensively studied, sex-specific effects on neurodevelopment remain poorly understood. Here we investigated the sex-dependent impact of maternal GDM on offspring brain development and behavior using a high-fat diet and low-dose streptozotocin induced mouse model. We found that adult female offspring exposed to maternal GDM exhibited depressive-like behaviors and sustained impairments in hippocampal neurogenesis across multiple developmental stages (embryonic, weaning and adult), characterized by reduced neural stem cell proliferation and altered differentiation. By contrast, male offspring displayed substantial metabolic dysfunction but no sustained neurogenic deficits beyond the embryonic period. Metabolomic analysis revealed persistent downregulation of myo-inositol in female offspring hippocampus, associated with disruptions in neurogenic signaling pathways. In vitro experiments with female-derived neural stem cells confirmed that hyperglycemic conditions directly impaired proliferation and differentiation, partly through oxidative stress mechanisms. These findings establish a sex-specific vulnerability to GDM-induced neurodevelopmental alterations and identify myo-inositol metabolism as a potential therapeutic target for preventing long-term neuropsychiatric consequences in female offspring.

**Figure:**
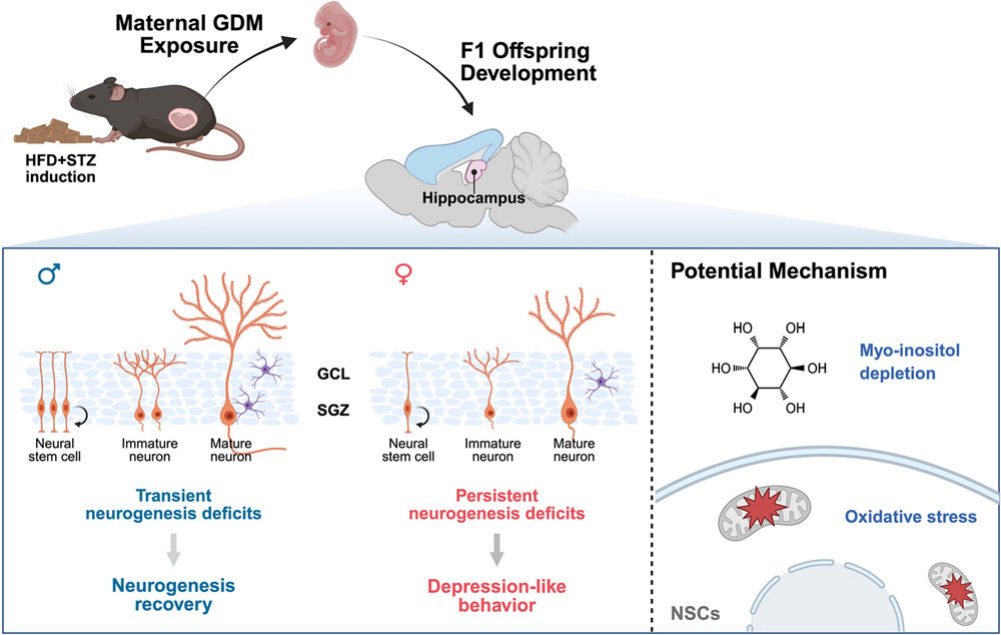
Maternal GDM induces sex-specific effects on offspring neurodevelopment, with females exhibiting persistent hippocampal neurogenesis deficits and depressive-like behaviors, while males show neurogenic resilience. The identification of myo-inositol depletion and oxidative stress as potential contributors to female-specific neurogenic impairments provides new insights into sex-specific vulnerability to maternal metabolic disturbances and suggests potential targets for intervention. HFD, High fat diet; STZ, Streptozotocin; GCL, Granule cell layer; SGZ, Subgranular zone; NSCs, Neural stem cells. Figure created with BioRender.com.

Maternal GDM induces sex-specific effects on offspring neurodevelopment, with females exhibiting persistent hippocampal neurogenesis deficits and depressive-like behaviors, while males show neurogenic resilience. The identification of myo-inositol depletion and oxidative stress as potential contributors to female-specific neurogenic impairments provides new insights into sex-specific vulnerability to maternal metabolic disturbances and suggests potential targets for intervention. HFD, High fat diet; STZ, Streptozotocin; GCL, Granule cell layer; SGZ, Subgranular zone; NSCs, Neural stem cells. Figure created with BioRender.com.

## Sex-specific effects of GDM on offspring neurogenesis

Gestational diabetes mellitus (GDM), a condition of glucose intolerance first identified during pregnancy, affects about 15% of pregnant women globally, posing risks to both maternal and offspring health. This study explores how GDM impacts neurodevelopment, particularly focusing on depressive- and anxiety-like behaviors in offspring. Using a GDM mouse model, researchers tracked hippocampal neurogenesis and examined neurometabolic changes and oxidative stress. The results showed that GDM selectively induces depressive-like behaviors in female offspring, linked to impaired neurogenesis and reduced synaptic plasticity in the hippocampus. Notably, female offspring exhibited decreased dendritic complexity and reduced levels of neurotransmitters such as serotonin and dopamine. These findings highlight the sex-specific impact of GDM on neurodevelopment, with potential implications for understanding the developmental origins of neuropsychiatric disorders. Future research could explore therapeutic interventions targeting metabolic-neural pathways to mitigate these effects.

This summary was initially drafted using artificial intelligence, then revised and fact-checked by the author.

## Introduction

Gestational diabetes mellitus (GDM), defined as glucose intolerance first diagnosed during pregnancy, affects approximately 15% of pregnant women worldwide^1,2^. GDM not only poses immediate risks to maternal health but also has long-term consequences for offspring, increasing their susceptibility to metabolic disorders such as obesity and type 2 diabetes (T2DM)^3,4^. Our previous work has similarly shown that maternal GDM predisposes offspring to a heightened risk of metabolic syndrome in adulthood^5^. Beyond metabolic dysfunction, there is growing evidence that GDM substantially impacts neurodevelopment and increases the risk of neuropsychiatric disorders in offspring^6–8^. Epidemiological studies suggest that children of mothers with GDM are at an elevated risk for conditions such as autism spectrum disorder (ASD) and attention-deficit hyperactivity disorder (ADHD)^9,10^. Notably, recent large cohort studies have revealed a dose-dependent relationship between maternal diabetes (T1DM > T2DM > GDM) and an increased vulnerability to depression and anxiety in offspring, highlighting hyperglycemia as a key modulator of neurobehavioral trajectories^11^.

The Developmental Origins of Health and Disease hypothesis proposes that prenatal and early postnatal adversities elevate the risk of diseases across the lifespan^12,13^. As the neural networks responsible for regulating emotions and behavior are primarily programmed during prenatal development, exposure to suboptimal intrauterine conditions may increase the likelihood of mental health disorders later in life^6,14^. The hippocampus, a brain region central to emotional regulation and stress resilience, is particularly vulnerable to such developmental programming^15^. Neural stem cells (NSCs) in the hippocampal dentate gyrus (DG) subgranular zone (SGZ) continuously self-renew and differentiate throughout life, contributing to the formation of functional neural circuits critical for emotional regulation^16,17^. Disruptions in this process can impair neurogenesis, which has been implicated in the pathophysiology of neuropsychiatric disorders^18–20^. However, whether and how GDM, as a prenatal stressor, induces long-term depressive- and anxiety-like behaviors in offspring via hippocampal neurogenic impairment remains poorly understood.

In this study, we established a GDM mouse model to investigate the effects of intrauterine hyperglycemia on depressive- and anxiety-like behaviors in adult offspring. We also longitudinally tracked hippocampal neurogenesis across developmental stages and explore the potential mechanisms underlying these behaviors, with a focus on neurometabolic changes and oxidative stress.

## Materials and methods

### Animals

Eight-week-old female C57BL/6J mice (18–22 g) were purchased from Hunan SJA Laboratory Animal Co. The mice were housed in a controlled environment at 22–24 °C with 40–60% humidity and a 12-h light/dark cycle. Mice were allowed to acclimatize for 1 week before the experiment, with free access to food and water throughout the study. All animal experiments were conducted in accordance with the guidelines of the Laboratory Animal Welfare and Ethics Code. All experimental protocols in this study were approved by the Animal Ethical and Welfare Committee of the First Affiliated Hospital of Chongqing Medical University (approval no. IACUC-2022-K410).

**Fig. 1:**
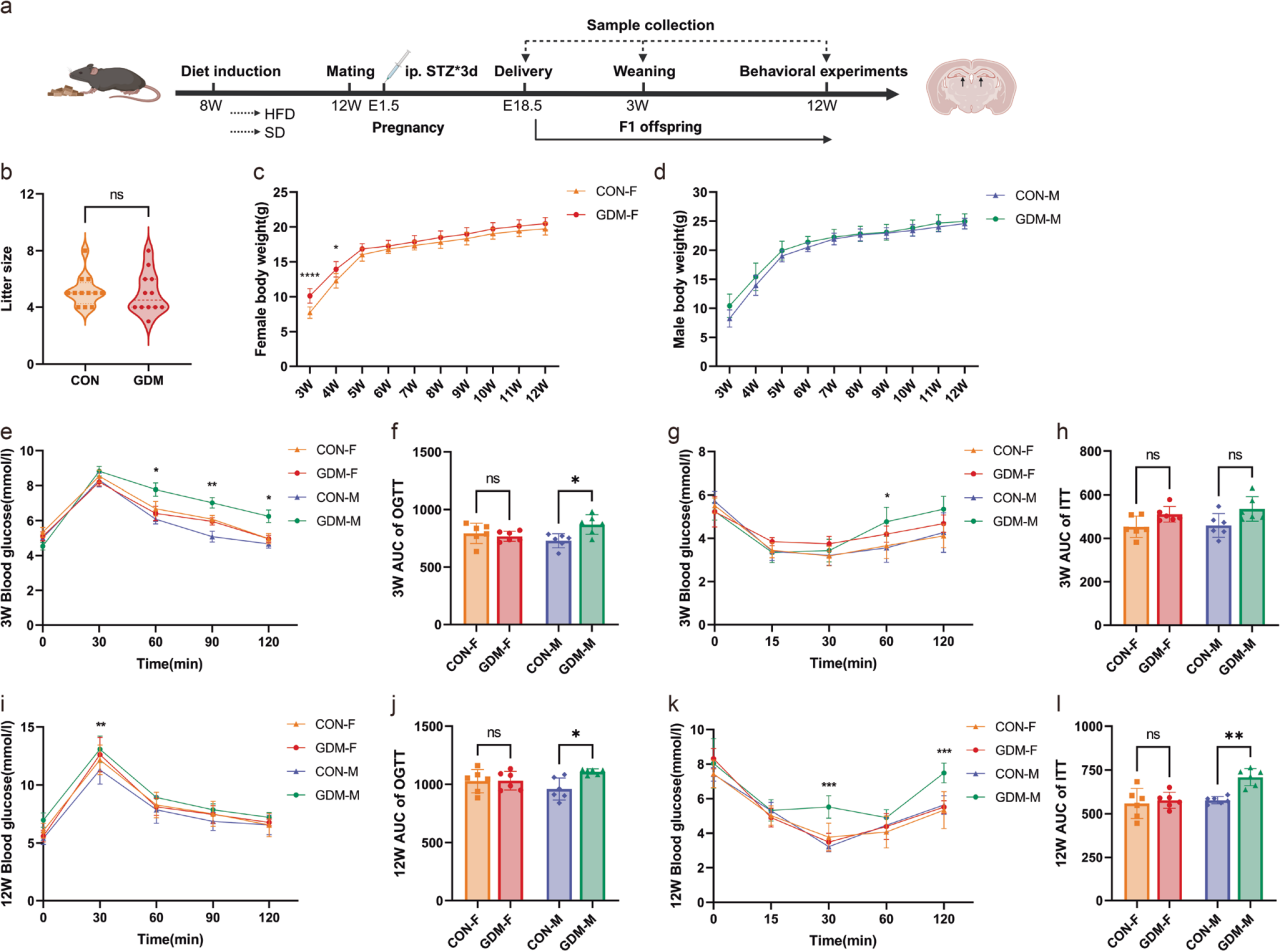
Maternal GDM induces metabolic alterations in male offspring. **a** A schematic of GDM model induction and experimental timeline. **b** Comparison of litter size between the GDM and control (CON) groups (*n* = 12). **c**, **d** Body weight growth curves for female (F; **c**) and male (M; **d**) offspring from weaning (3 W) to adult (12 W) stage (*n* = 12). **e**, **f** OGTT curves (**e**) and area under the curve (AUC) (**f**) comparison at weaning (*n* = 6). **g**, **h** ITT curves (**g**) and AUC comparison (**h**) at weaning (*n* = 6). **i**, **j** OGTT curves (**i**) and AUC comparison (**j**) in adult offspring (*n* = 6). **k**, **l** ITT curves (**k**) and AUC comparison (**l**) in adult offspring (*n* = 6). Values are represented as mean ± s.d. Statistical tests were assessed by an unpaired Student’s *t*-test or two-way ANOVA. For longitudinal data, a two-way repeated measures ANOVA was used, followed by Tukey’s post hoc test to assess differences at each time point. *P < 0.05, **P < 0.01, ***P < 0.001, ****P < 0.0001, indicating a significant difference between GDM and control groups of the same sex. ns, not significant.

Female C57BL/6J mice were randomly assigned to either the GDM group or the control group. The GDM group was fed a high-fat diet (HFD; D12492, Research Diet), while the control group was fed a standard diet. After 4 weeks of dietary induction, female mice were mated with male mice at a 2:1 ratio. Pregnancy was confirmed by the presence of a vaginal plug, which was designated as embryonic day 0.5 (E0.5). From E1.5, the GDM group received intraperitoneal injections of low-dose streptozotocin (STZ; 30 mg/kg, S0130, Sigma) for 3 consecutive days, while the control group received an equivalent volume of citrate buffer. Random blood glucose levels were monitored at E4.5, E7.5 and E10.5 via tail vein blood samples using a glucometer (Roche). At E16.5, an oral glucose tolerance test (OGTT) and an insulin tolerance test (ITT) were performed. Successful establishment of the GDM model was confirmed when random blood glucose levels exceeded 11.1 mmol/l (ref. ^21^). Pregnant mice were allowed to deliver naturally, and the litter size was randomly reduced to six pups at birth to ensure uniform nutrition. The F1 offspring were weaned at 3 weeks, and behavioral testing was performed at 12 weeks. Hippocampal samples from F1 offspring were collected at E18.5, 3 weeks and 12 weeks for subsequent analysis (Fig. 1a).

### Glucose tolerance test

The OGTT was performed on pregnant mice at E16.5 and on F1 adult offspring at 12 weeks. The mice were fasted for 8 h with free access to water. Before the experiment, the mice were weighed and fasting blood glucose was measured. A 20% D-glucose solution (2 g/kg body weight) was administered via oral gavage and blood glucose levels were measured at 30, 60, 90 and 120 min post-gavage via tail vein blood collection.

For the intraperitoneal glucose tolerance test (ipGTT), 3-week-old offspring mice were fasted for 6 h with free access to water. The mice were intraperitoneally injected with a 20% D-glucose solution (1 g/kg body weight). The remaining procedures were the same as those for the OGTT.

### ITT

After a 6-h fast, fasting blood glucose was measured. Pregnant mice at E16.5 and adult F1 offspring were intraperitoneally injected with insulin (1 U/kg), while 3-week-old F1 offspring received 0.75 U/kg. Blood glucose levels were measured at 0, 15, 30, 60 and 120 min postinjection.

### Behavioral tests

#### OFT

The open field tests (OFTs) were conducted on 12-week-old F1 offspring, separated by sex. Mice were acclimatized to the testing room for 3 h in a quiet, low-light environment before testing. During the test, each mouse was placed in the center of the open field apparatus (40 cm × 40 cm × 40 cm)^22^. Activity was automatically tracked for 5 min using ANY-maze software (Stoelting).

#### EPM

For the elevated plus maze test (EPM), each mouse was placed in the center of the EPM, oriented toward an open arm. Mouse movement was recorded automatically for 5 min using ANY-maze software. The following indicators were analyzed: total distance traveled, percentage of entries into open arms (OE%) and percentage of time spent in open arms (OT%)^22^.

#### EZM

For the elevated zero maze test (EZM), mice were placed in the center of the EZM, and movement was tracked and recorded automatically for 5 min using ANY-maze software. The total distance traveled, OE% and OT% were analyzed^23^.

#### FST

For the forced swim test (FST), following a 3-h acclimation period, mice were placed individually in a glass cylinder (30 cm in height, 20 cm in diameter) filled with 15 cm of water (23–25 °C). Each session consisted of a 2-min adaptation period in the apparatus, immediately followed by a 5-min test period during which movements were recorded. After the test period, mice were gently dried with towels and placed in a dry recovery cage on a heating pad to maintain body temperature. Animals were monitored until normal respiration and locomotion were observed before being returned to their original cage. The duration of immobility, swimming and struggling time were automatically quantified using ANY-maze software. For the analysis, data from the entire 5-min test period and initial 4 min of the recording were used^24^.

#### TST

For the tail suspension test (TST), after a 3-h acclimation period, mice were suspended by the tail from a horizontal bar using 17 cm adhesive tape. The suspension height was approximately 20 cm from the floor. Each session consisted of a 2-min adaptation period in the apparatus, immediately followed by a 5-min test period during which movements were recorded. The duration of immobility and mobility time were automatically quantified using ANY-maze software. For the analysis, data from the entire 5-min test period and initial 4 min of the recording were used^25^.

#### SPT

For the sucrose preference test (SPT), mice were housed individually in cages, each containing two sealed water bottles. During a 3-day acclimatization period, the positions of the water bottles were swapped daily to minimize side bias. During testing, one bottle was replaced with a 1% sucrose solution, while the other contained pure water. The amount of water consumed from each bottle was measured and sucrose preference was calculated as follows: sucrose preference (%) = (sucrose solution consumption/(sucrose solution + water consumption)) × 100% (ref. ^26^).

### RT–qPCR

Total RNA was extracted from hippocampal tissue using Trizol reagent (Invitrogen). Complementary DNA (cDNA) was synthesized using the PrimeScript RT Reagent kit with gDNA Eraser (HY-K0511, MCE). RT–qPCR was performed using Green SYBR Premix Ex Taq II (HY-K0521, MCE) on the CFX96 Real-Time System (Bio-Rad). Primers are listed in Supplementary Table 1.

### DA and 5-HT measurement

Hippocampal tissues were washed with prechilled PBS, weighed and then homogenized thoroughly. The homogenate was centrifuged at 5,000*g* for 10 min at 2–8 °C and the supernatant was collected for further analysis. The concentrations of dopamine (DA) and 5-hydroxytryptamine (5-HT) in the tissue samples were measured using enzyme-linked immunosorbent assay (ELISA) kits (E-EL-0046/E-EL-0033, Elabscience).

### Histological staining

#### Sample collection and hematoxylin-eosin staining

After behavioral testing, mice were anesthetized by intraperitoneal injection of sodium pentobarbital (50 mg/kg) and transcardially perfused with 0.9% saline followed by 4% paraformaldehyde (PFA). The paraffin-embedded sections were deparaffinized in xylene, rehydrated through a graded ethanol series and stained with hematoxylin for 5–10 min. After washing, the sections were counterstained with eosin for 2–3 min. Following staining, the sections were dehydrated, cleared in xylene and sealed with resinene.

#### Glycine silver staining

Tissue sections were deparaffinized and rehydrated through a graded alcohol series. Sections were then incubated in a 0.25% silver nitrate solution (G1052, Servicebio) for 30 min at room temperature, followed by incubation in a glycine-formaldehyde solution for 2 h to enhance silver deposition. After staining, the sections were washed in distilled water, dehydrated through increasing concentrations of alcohol, cleared in xylene and sealed with resinene.

#### Bielschowsky’s silver staining

Paraffin-embedded hippocampal tissues were cut at 10 μm, deparaffinized and rehydrated. The staining was performed using a commercial kit (G3260, Solarbio) according to the manufacturer’s instructions. Sections were first incubated in a pretreatment solution for 30 min at 37 °C. After rinsing, sections were impregnated with an ammoniacal silver solution, followed by development in a reducing solution to visualize neuronal structures. The reaction was then stopped with a thiosulfate solution and sections were toned to enhance contrast. Finally, sections were dehydrated through a graded ethanol series, cleared in xylene and sealed with a resinous mounting medium.

#### Golgi–Cox staining

Fresh hippocampal tissue was fixed in 4% PFA for 24–48 h. After fixation, the tissue was incubated in Golgi–Cox solution (GP1152, Servicebio) for 2 weeks at room temperature in the dark, with the solution being replaced every 2–3 days to ensure optimal impregnation. After incubation, the tissue was sectioned at 60-μm thickness using a vibratome and sections were mounted on slides. Sections were incubated in a developer solution for 30 min, washed in distilled water and sealed with glycerin gelatin. Dendritic spine density and complexity were assessed using light microscopy.

#### BrdU labeling

Pregnant dams received intraperitoneal injections of 5-bromo-2′-deoxyuridine (BrdU; B8010, Solarbio) for 3 consecutive days (100 mg/kg/day) at E12.5. F1 offspring were similarly intraperitoneally injected with BrdU for 3 consecutive days at 2 weeks of age or 11 weeks of age. After injections, hippocampal tissues from F1 offspring were collected at E18.5, 3 weeks and 12 weeks for fixation, dehydration, paraffin embedding and sectioning.

#### Immunofluorescence staining

Paraffin-embedded hippocampal sections were deparaffinized and rehydrated. Antigen retrieval was performed using EDTA antigen retrieval buffer in a microwave for 10 min. Cell slides were fixed with 4% PFA for 10 min and then permeabilized with 0.1% Triton X-100 for 1 h. Blocking was performed by incubating the tissue sections or cell coverslips with 1% BSA for 30 min. Primary antibodies were applied overnight at 4 °C. The following primary antibodies were used: mouse anti-BrdU (1:500, HA601311, HUABIO), rabbit anti-Nestin (1:200, A11861, ABclonal), rabbit anti-Doublecortin (DCX; 1:500, HA601398, HUABIO), rabbit anti-NeuN (1:200, ET1602-12, HUABIO), rabbit anti-GFAP (1:500, EM140707, HUABIO) and rabbit anti-TOMM20 (1:200, ET1609-25, HUABIO). The following day, sections were incubated with secondary antibodies (1:1000, Beyotime) at room temperature for 1 h. After washing with PBS, sections were stained with DAPI (G1012, Servicebio) for 5 min at room temperature to counterstain the nuclei. The sections were mounted with antifluorescence quenching mounting medium and examined under a fluorescence microscope.

#### Primary NSCs isolation and culture

Primary NSCs were isolated from the hippocampi of E12.5 mouse embryos. The hippocampi were dissected in prechilled PBS under a dissecting microscope. The tissue was then mechanically triturated to a single-cell suspension in complete medium. This complete medium was composed of KnockOut DMEM/F-12 (96%, 12660012, Gibco), GlutaMAX (1%, 35050061, Gibco), B27 (2%, 12587010, Gibco), bFGF (20 µg/L, 450-33, PeproTech), EGF (20 µg/L, 315-09, PeproTech), heparin (2 µg/mL, 9041-08-1, Solarbio) and penicillin/streptomycin (1%, C0222, Beyotime). The cell suspension was filtered through a 70-μm mesh filter and centrifuged at 100*g* for 5 min. The cell pellet was resuspended and seeded at 1 × 10^6^ cells/mL in T25 flasks. Cells were cultured at 37 °C in a 5% CO_2_ incubator, forming neurospheres within 3–5 days. Half of the medium was replaced with fresh complete medium every 2–3 days, and neurospheres were passaged every 6-7 days (refs. ^27,28^).

#### In vitro hyperglycemia modeling

After passaging and seeding, primary NSCs were first cultured in complete medium for 6 h to allow for cell recovery and attachment. The medium was then replaced with complete medium prepared using a low-glucose DMEM base (1 g/L glucose; C11885500BT, Gibco) and cells were cultured overnight. Following this synchronization, hyperglycemia was simulated by supplementing the medium with sterile D-glucose to the final concentrations for the duration of the experiment.

#### CCK-8 assay

Primary NSCs were seeded at a density of 2 × 10^5^ cells/mL into 96-well plates. Then 10 μL CCK-8 reagent (C0039, Beyotime) was added to each well. After incubation at 37 °C for 2 h, the absorbance at 450 nm was measured using a microplate reader (Bio-Rad). The absorbance values were compared for treatment with different glucose concentrations from day 0 to day 4.

#### NSCs induction and differentiation

Twelve-well plates were pre-coated with 0.1% poly-L-lysine solution (ST509, Beyotime) for 24 h at 37 °C. Primary NSCs were separated into single-cell suspensions at a density of 2 × 10^5^ cells/mL and seeded onto coated plates. Cells were cultured in differentiation medium, supplemented with 1% FBS (A5670701, Gibco) and without bFGF, EGF and heparin. On day 7 of differentiation induction, the cells were fixed with prechilled 4% PFA for immunofluorescence staining^28,29^.

#### Nontargeted metabolomic analysis

Metabolomic analysis was performed by Shanghai OE Biotech Co. Briefly, 30 mg of hippocampal tissue was homogenized in 600 μL methanol–water (4:1, v/v) containing the internal standard. After ultrasonic extraction and centrifugation, the supernatant was collected and dried. The samples were derivatized with methoxyamine hydrochloride and BSTFA before gas chromatography–mass spectrometry (GC–MS) analysis.

Chromatographic separation was carried out using a DB-5MS capillary column (Agilent J&W Scientific) with helium as the carrier gas. Mass spectrometry was performed in electron impact ionization mode (70 eV), with a scan range of *m*/*z* 50–500. Data were processed using MS-DIAL software for peak detection, identification, alignment and normalization. Metabolites were identified by matching the retention times and fragment ion mass spectra (EI fragmentation patterns) against a self-built database (LuMet-GC 5.0, Lumingbio). Differential metabolites were selected based on *P* < 0.05 and fold change (FC) ≥1.2 or ≤1/1.2.

#### ROS, GSH and NAD^+^ detection

Intracellular reactive oxygen species (ROS) levels were measured using the DCFH-DA probe (S0033S, Beyotime). For the quantification of glutathione (GSH) and NAD⁺/NADH ratios, commercial colorimetric assay kits were used (S0053/S0175, Beyotime). All procedures were performed following the manufacturer’s instructions. The absorbance was measured using a microplate reader (Bio-Rad) and the final values were normalized to the total protein concentration of each sample.

#### Mitochondrial membrane potential assay

The mitochondrial membrane potential was evaluated using a JC-1 Kit (C2006, Beyotime). Treated primary NSCs were incubated with JC-1 solution at 37 °C for 20 min and then detected using a fluorescence microscope. A decrease in the red/green fluorescence intensity ratio indicates mitochondrial depolarization.

#### Statistical analysis

Data were analyzed using GraphPad Prism software (version 9.5) and are presented as the mean ± s.d. For comparisons between two independent groups, a two-tailed unpaired Student’s *t*-test was used. For experiments involving two independent factors (treatment and sex), a two-way analysis of variance (ANOVA) was used to assess main effects and interactions followed by Tukey’s post hoc test. For longitudinal data, a two-way repeated measures ANOVA was used, followed by Tukey’s post hoc test to assess differences at each time point. *P* < 0.05 was considered statistically significant.

## Results

### GDM induces metabolic alterations in maternal and male offspring

To investigate the developmental impact of GDM on offspring, we established a combined HFD and low-dose streptozotocin mouse model. Dams exposed to a 4-week pregestational HFD showed moderate weight gain (HFD: 22.48 ± 1.07 g versus standard diet: 21.16 ± 1.23 g; *P* < 0.05), without developing obesity or hyperglycemia (Supplementary Fig. 1a,b). Longitudinal fasting blood glucose monitoring revealed progressive hyperglycemia in GDM dams during mid-gestation, with significant elevations at E7.5 and E10.5 (Supplementary Fig. 1c). By late gestation (E16.5), impaired glucose tolerance and insulin resistance confirmed successful model establishment (Supplementary Fig. 1d–g).

Sex-specific metabolic programming in offspring was further examined. Litter size analysis showed no significant differences between the groups (Fig. 1b). Body weight trajectories exhibited transient growth acceleration in both sexes of GDM offspring during early postnatal development, with normalization by adulthood (Fig. 1c, d). Assessments of glucose homeostasis revealed sex-specific metabolic outcomes. At weaning, male GDM offspring displayed significant glucose intolerance, whereas females maintained normal glucose responses (Fig. 1e, f). ITT at this stage showed no intergroup differences in either sex (Fig. 1g,h). In adulthood, metabolic dysfunction persisted exclusively in males, characterized by exacerbated glucose intolerance (Fig. 1i, j) and emerging insulin resistance (Fig. 1k, l). These findings demonstrate that maternal GDM selectively programs male offspring for lifelong glycometabolic dysfunction, potentially increasing their risk of developing metabolic diseases in adulthood.

### GDM offspring do not exhibit anxiety-like behavior regardless of sex

**Fig. 2:**
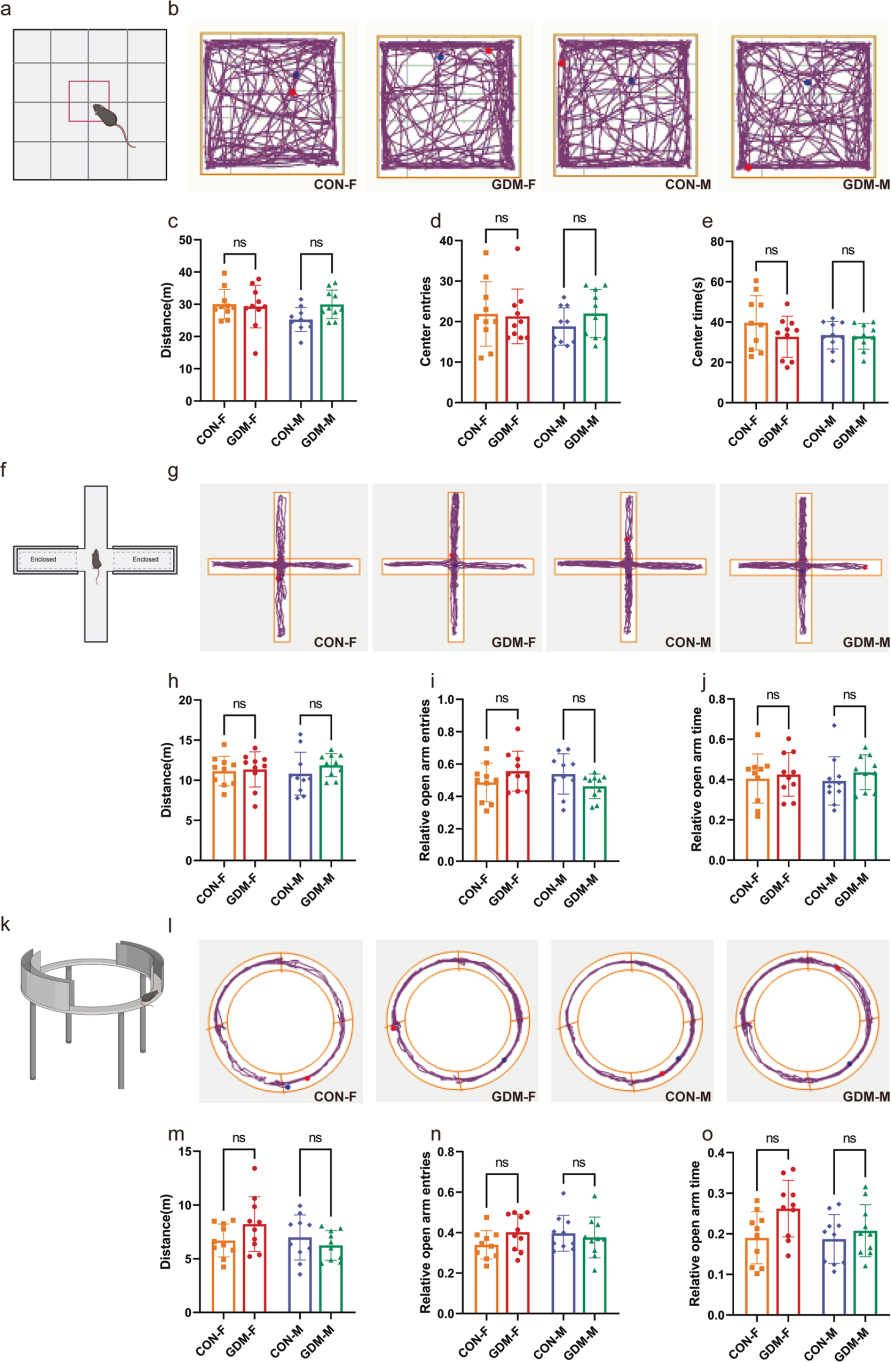
Maternal GDM does not induce anxiety-like behaviors in adult offspring. **a** A schematic of the OFT. **b** Representative movement trajectories in the OFT. **c**–**e** Quantification of total distance traveled (**c**), number of entries into the center zone (**d**) and time spent in the center zone (**e**). **f** A schematic of the EPM. **g** Representative movement trajectories in the EPM. **h**–**j** Quantification of total distance traveled (**h**), percentage of entries into open arms (**i**) and percentage of time spent in open arms (**j**). **k** A schematic of the EZM. **l** Representative movement trajectories in the EZM. **m**–**o** Quantification of total distance traveled (**m**), percentage of entries into open arms (**n**) and percentage of time spent in open arms (**o**). Values are presented as mean ± s.d. (*n* = 10 per group). Statistical significance was assessed by a two-way ANOVA with Tukey’s post hoc test. No significant differences were found between any of the groups. ns, not significant.

To assess the long-term behavioral consequences of GDM, we performed a series of sex-stratified behavioral tests on 12-week-old offspring. Anxiety-like behaviors were evaluated using the OFT, EPM and EZM. In the OFT, no significant differences were observed between the GDM and control groups in locomotor activity or exploratory behavior, as measured by distance, center entries and center time (Fig. 2a–e). Similarly, in the EPM and EZM, there were no significant differences between the GDM and control groups in total activity (Fig. 2f–h, k–m), open arm entries (Fig. 2i, n) or time spent in open arms (Fig. 2j, o). These findings collectively indicate that maternal GDM does not induce anxiety phenotypes in offspring.

### GDM induce depressive-like behavior specifically in female offspring

**Fig. 3:**
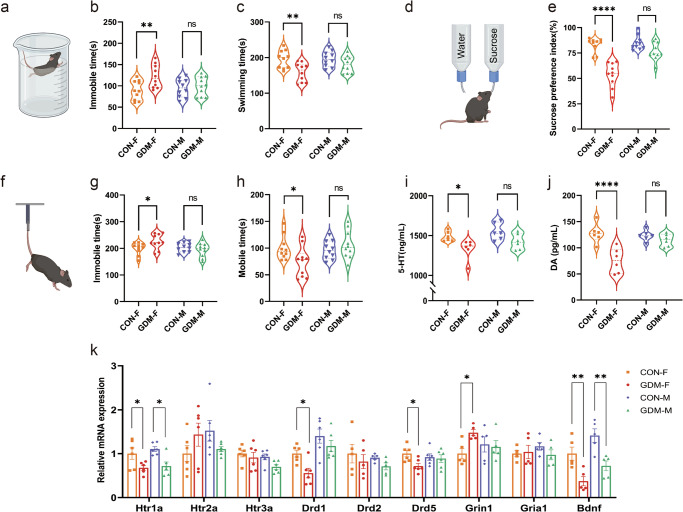
Maternal GDM induces depressive-like behaviors in adult female offspring. **a** A schematic of the FST. **b**, **c** Quantification of immobility time (**b**) and swimming time (**c**) (*n* = 10). **d** A schematic of the SPT. **e** The sucrose preference ratio (*n* = 10). **f** A schematic of the TST. **g**, **h** Quantification of immobility time (**g**) and mobility time (**h**) (*n* = 10). **i**, **j** Hippocampal serotonin (5-HT) (**i**) and DA (**j**) levels measured by ELISA (*n* = 6). **k** Relative mRNA expression of 5-HT receptors, DA receptors, glutamate receptor and BDNF in the hippocampus (*n* ≥ 5). Values are presented as mean ± s.e.m. Statistical significance was assessed by a two-way ANOVA with Tukey’s post hoc test. *P < 0.05, **P < 0.01, ***P < 0.001, ****P < 0.0001, indicating a significant difference between GDM and control groups of the same sex. ns, not significant.

Depressive-like behaviors in offspring were assessed using the FST, SPT and TST. In the FST, GDM female offspring exhibited a significant increase in immobility time compared with controls, along with a corresponding decrease in swimming duration, indicating behavioral despair. By contrast, no significant differences were observed in males (Fig. 3a–c and Supplementary Fig. 2a,b). In the SPT, GDM female offspring displayed a significant reduction in sucrose preference, indicating anhedonia, while males maintained normal hedonic responses (Fig. 3d, e). The TST also showed a significant increase in immobility time and a decrease in struggling time in GDM female offspring, with no significant differences observed in males, reinforcing the previous findings (Fig. 3f–h and Supplementary Fig. 2c,d).

To further investigate the impact of GDM on depression in female offspring, we analyzed hippocampal tissues for neurotransmitter systems and neurotrophic factors using ELISA and RT–qPCR. The results showed reduced 5-HT and DA levels in GDM female offspring, with no changes in males (Fig. 3i,j). In addition, RT–qPCR analysis demonstrated downregulation of 5-HT receptor 1A (Htr1a), DA receptor D1 (Drd1) and D5 (Drd5), alongside upregulated glutamate receptor ionotropic NMDA1 (Grin1) expression in GDM female offspring (Fig. 3k). Brain-derived neurotrophic factor (BDNF) expression was suppressed in both sexes (Fig. 3k). These results indicate that maternal GDM induces sex-specific depressive phenotypes in female offspring.

### GDM leads to structural adaptations in both sex offspring hippocampus

**Fig. 4:**
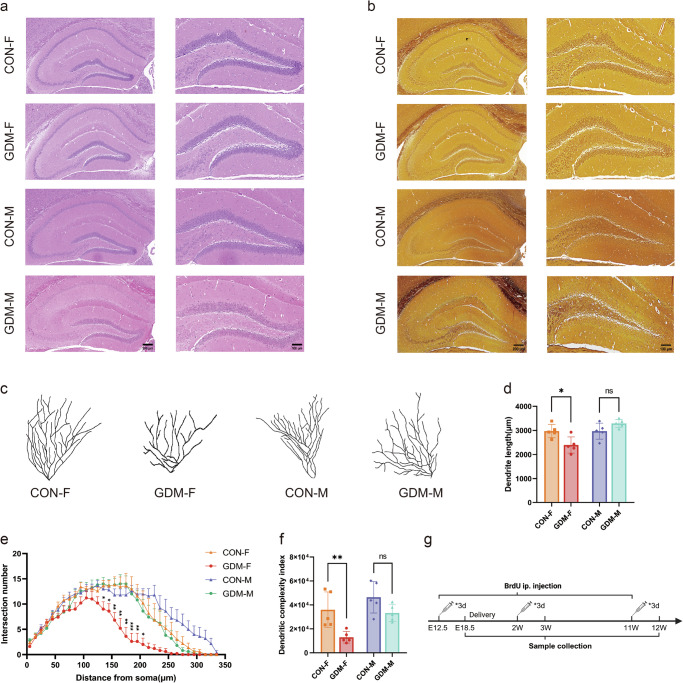
GDM offspring exhibit structural adaptations but impaired synaptic plasticity in females. **a**, **b** Representative images of H&E staining (**a**) and silver staining (**b**) of adult hippocampal tissue. **c** Representative reconstruction of Golgi–Cox stained neurons from hippocampal DG region. **d** Quantification of dendritic length (*n* = 5). **e** Sholl analysis of dendritic branching complexity (*n* = 5). **f** Analysis of the dendritic complexity index (*n* = 5). **g** A schematic timeline of BrdU administration and tissue collection for the neurogenesis experiments. Values are represented as mean ± s.d. Statistical significance was assessed by a two-way ANOVA with Tukey’s post hoc test. *P < 0.05, **P < 0.01, indicating a significant difference between GDM and control groups of the same sex. ip, intraperitoneal; ns, not significant.

The hippocampus plays a crucial role in mood regulation and is strongly implicated in the development of depression. Therefore, hippocampal tissue from adult offspring was stained to assess potential structural alterations. H&E staining revealed preserved cytoarchitecture in both male and female GDM offspring, with normal neuronal morphology, organized neuronal layers and intact nuclear features, similar to controls (Fig. 4a). Furthermore, glycine silver and Bielschowsky’s silver staining confirmed the absence of degenerative changes. Both stains showed normal neuronal process orientation and density in hippocampal subregions (CA1, CA3 and DG), and critically, no pathological hallmarks such as argyrophilic plaques or neurofibrillary tangles were observed in any GDM offspring (Fig. 4b and Supplementary Fig. 3a). These findings suggest that maternal GDM does not impair hippocampal structural integrity in either male or female offspring.

### GDM selectively reduces synaptic plasticity in female offspring hippocampus

To further examine synaptic structure changes, we performed Golgi–Cox staining of hippocampal neurons. Representative images of hippocampal neurons showed reduced dendritic arborization in GDM female offspring compared with controls, while males exhibited no structural alterations (Fig. 4c). Quantitative analyses revealed a significant reduction in dendritic length in GDM female offspring, with no changes in males (Fig. 4d). Sholl analysis showed a significant reduction in the number of dendritic branches in GDM female offspring compared with controls, whereas males showed no difference (Fig. 4e). Statistical analysis revealed a significant reduction in dendritic complexity in GDM female offspring (Fig. 4f). These results indicate that maternal GDM impairs synaptic plasticity specifically in female offspring, while male offspring remain unaffected.

### GDM impairs adult hippocampal neurogenesis exclusively in female offspring

Decreased adult neurogenesis in the hippocampus is a well-established contributor to depression. To investigate whether this mechanism underlies the depressive-like phenotype in GDM offspring, we first examined the total population of mature neurons. Quantification of NeuN-positive cells revealed a significant reduction in the neuronal density specifically within the DG region of GDM female offspring (Supplementary Fig. 3b,e). This deficit was not observed in males and did not reach statistical significance in the non-neurogenic CA1 and CA3 regions of females (Supplementary Fig. 3b–e).

**Fig. 5:**
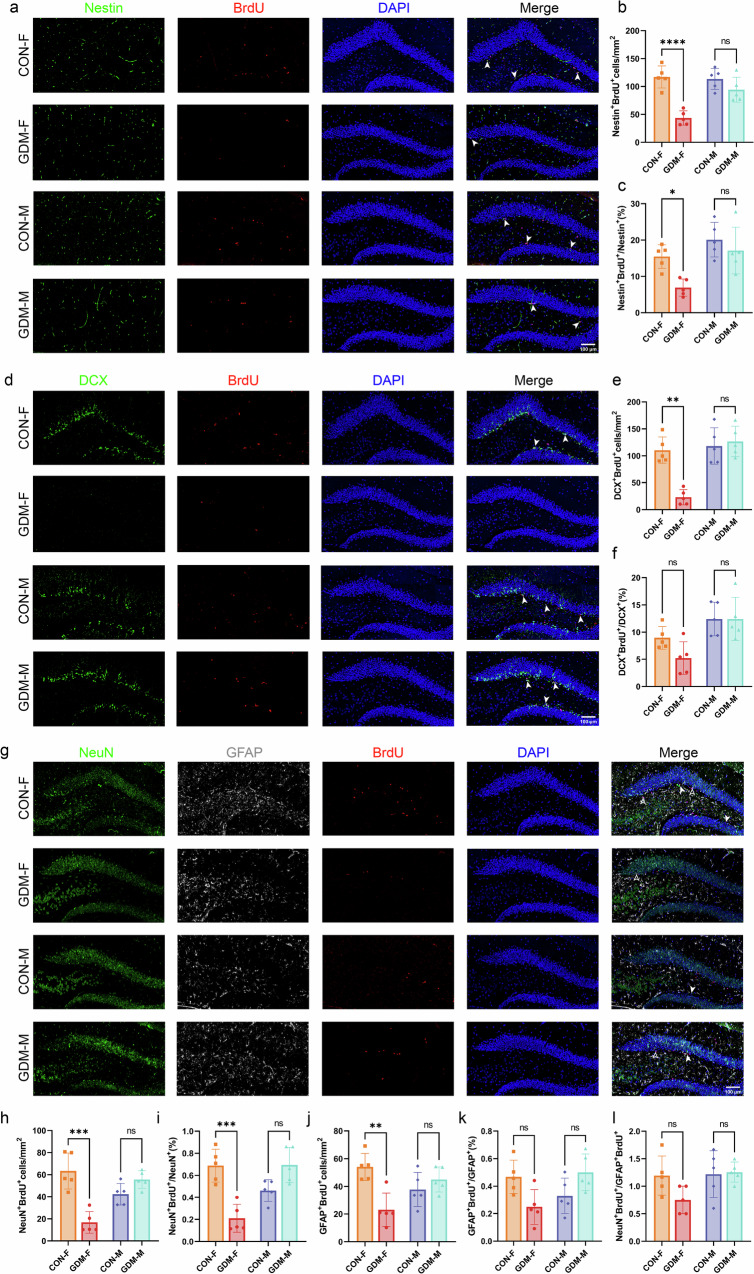
Maternal GDM reduces hippocampal neurogenesis in adult female offspring. **a** Representative images of BrdU^+^ (red) and Nestin^+^ (green) cells in the DG region of adult offspring (12 W). Solid white arrows indicate Nestin^+^BrdU^+^ co-labeled cells. **b** Quantification of the number of Nestin^+^BrdU^+^ cells. **c** The ratio of newly generated Nestin^+^ cells. **d** Representative images of BrdU^+^ (red) and DCX^+^ (green) cells in the DG region. Solid white arrows indicate DCX^+^BrdU^+^ co-labeled cells. **e** Quantification of the number of DCX^+^BrdU^+^ cells. **f** The ratio of newly generated DCX^+^ cells. **g** Representative images of BrdU^+^ (red), NeuN^+^ (green) and GFAP^+^ (gray) cells in the DG region. Solid white arrows indicate NeuN^+^BrdU^+^ co-labeled cells, while the hollow white arrow points to GFAP^+^BrdU^+^ co-labeled cells. **h** Quantification of the number of NeuN^+^BrdU^+^ cells. **i**, The ratio of newly generated NeuN^+^ neurons. **j** Quantification of the number of GFAP^+^BrdU^+^ cells. **k** The ratio of newly generated GFAP^+^ astrocytes. **l** The ratio of newly generated neurons (NeuN^+^BrdU^+^) to astrocytes (GFAP^+^BrdU^+^). Values are presented as mean ± s.d. (*n* = 5 per group). Statistical significance was assessed by a two-way ANOVA with Tukey’s post hoc test. *P < 0.05, **P < 0.01, ***P < 0.001, ****P < 0.0001, indicating a significant difference between GDM and control groups of the same sex. ns, not significant.

Having identified a deficit in the mature neuron pool, we next sought to determine whether it resulted from an impaired neurogenic process. To this end, we administered BrdU injections to 11-week-old offspring to label dividing cells and subsequently tracked their fate (Fig. 4g). The proliferation, differentiation and maturation of NSCs were assessed by co-labeling with Nestin, DCX, NeuN and GFAP. At 12 weeks, GDM female offspring exhibited reduced NSC proliferation, with significantly fewer BrdU^+^Nestin^+^ cells and a lower proliferation ratio compared with controls in the DG region, while males showed no differences (Fig. 5a–c). Neuronal differentiation was also impaired in females, as evidenced by fewer BrdU^+^DCX^+^ immature neurons in the DG region, while males remained unaffected (Fig. 5d–f). Furthermore, the maturation of newborn neurons was disrupted in GDM female offspring, with fewer BrdU^+^NeuN^+^ mature neurons and a reduced maturation ratio, whereas no such differences were observed in males (Fig. 5g–i). In addition, astrocytic differentiation analysis revealed fewer BrdU^+^GFAP^+^ cells in GDM females, but the differentiation ratio remained unchanged, with no differences in males (Fig. 5j,k). The ratio of newly generated mature neurons to astrocytes showed no imbalance in NSC differentiation between the GDM and control groups (Fig. 5l). These results suggest that maternal GDM selectively impairs adult hippocampal neurogenesis in female offspring, characterized by suppressed proliferation and differentiation of NSCs, without disrupting differentiation balance.

### GDM affects early hippocampal neurogenesis with persistent deficits in female offspring

**Fig. 6:**
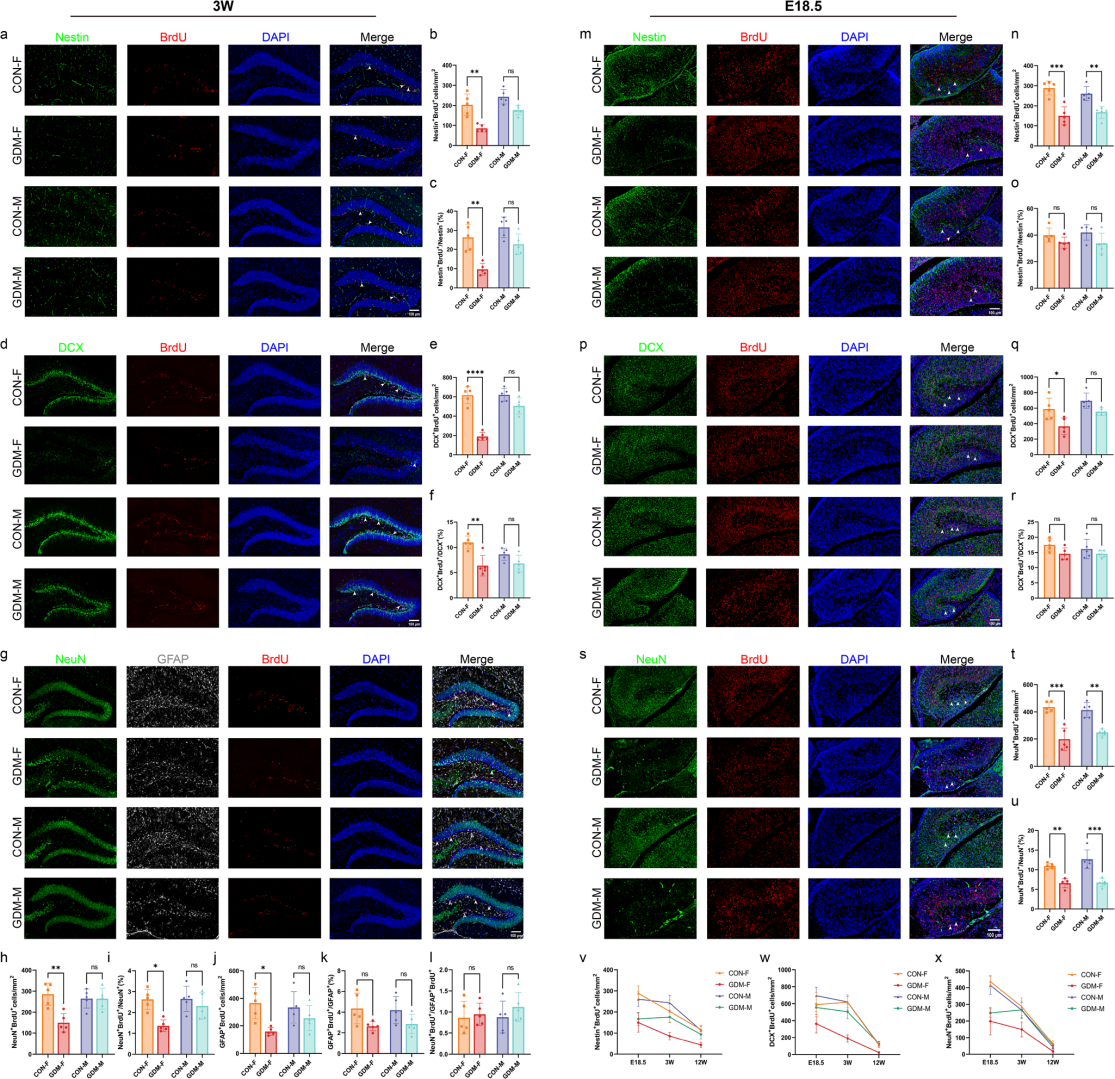
Maternal GDM reduces early hippocampal neurogenesis in female offspring. **a**–**u** Hippocampal neurogenesis was assessed at the weaning stage (**a**–**l**) and the embryonic stage (**m**–**u**). **a** At weaning (3 W), representative images of BrdU^+^ (red) and Nestin^+^ (green) cells in the DG region. Solid white arrows indicate Nestin^+^BrdU^+^ co-labeled cells. **b** Quantification of the number of Nestin^+^BrdU^+^ cells. **c** The ratio of newly generated Nestin^+^ cells. **d** Representative images of BrdU^+^ (red) and DCX^+^ (green) cells in the DG region. Solid white arrows indicate DCX^+^BrdU^+^ co-labeled cells. **e** Quantification of the number of DCX^+^BrdU^+^ cells. **f**, The ratio of newly generated DCX^+^ cells. **g** Representative images of BrdU^+^ (red), NeuN^+^ (green) and GFAP^+^ (gray) cells in the DG region. Solid white arrows indicate NeuN^+^BrdU^+^ co-labeled cells, while the hollow white arrow points to GFAP^+^BrdU^+^ co-labeled cells. **h** Quantification of the number of NeuN^+^BrdU^+^ cells. **i** The ratio of newly generated NeuN^+^ neurons. **j** Quantification of the number of GFAP^+^BrdU^+^ cells. **k** The ratio of newly generated GFAP^+^ astrocytes. **l** The ratio of newly generated neurons (NeuN^+^BrdU^+^) to astrocytes (GFAP^+^BrdU^+^). m–u, At the embryonic stage (E18.5), a similar set of analyses was performed. **m–o** Representative images **(m)**, quantification of co-labeled cells **(n),** and the ratio of newly generated cells **(o)** for NSC proliferation (BrdU^+^Nestin^+^). p–r Representative images **(p)**, quantification **(q)**, and ratio **(r)** for differentiation into immature neurons (BrdU^+^DCX^+^). s–u Representative images **(s)**, quantification **(t)**, and ratio **(u)** for maturation into new neurons (BrdU^+^NeuN^+^). **v**–**x** The developmental trajectories of NSCs proliferation **(v)**, neuronal differentiation **(w)** and maturation **(x)** in the hippocampus. Values are presented as mean ± s.d. (*n* = 5 per group). Statistical significance was determined by a two-way ANOVA with Tukey’s post hoc test. *P < 0.05, **P < 0.01, ***P < 0.001, ****P < 0.0001, indicating a significant difference between GDM and control groups of the same sex. ns, not significant.

Maternal GDM alters the intrauterine environment, exerting long-lasting effects on offspring neurogenesis. To investigate the developmental impacts, we administered BrdU to E12.5 dams and 2-week-old offspring to label proliferating cells and assessed NSC dynamics across early developmental stages (Fig. 4g). At weaning (3 weeks), GDM female offspring exhibited significant reductions in BrdU^+^Nestin^+^ cells (Fig. 6a–c) and BrdU^+^DCX^+^ immature neurons (Fig. 6d–f), while males showed no significant changes. Furthermore, GDM female-specific impairments extended to BrdU^+^NeuN^+^ mature neurons generation and BrdU^+^GFAP^+^ astrocytic differentiation, though differentiation balance remained unaltered (Fig. 6g–l).

At E18.5, the number of BrdU^+^Nestin^+^ cells was reduced in both sexes, but there was no impact on the proliferation ratio (Fig. 6m–o). In addition, the number of BrdU^+^DCX^+^ cells was selectively impaired in GDM females, with the proportion unchanged, while males showed no deficits (Fig. 6p–r). Since NSC differentiation during embryogenesis primarily favors neuronal differentiation, we focused on analyzing newly generated mature neurons. The results showed that the number of BrdU^+^NeuN^+^ mature neurons was globally suppressed in both sexes, with reduced maturation ratios (Fig. 6s–u). Finally, we performed a temporal analysis of NSC proliferation, differentiation and maturation (Fig. 6v–x), confirming that the effects of maternal GDM on offspring NSCs originated in the intrauterine environment and persisted postnatally. Notably, while both sexes exhibited neurogenic deficits during embryonic development, only females showed persistent impairments through weaning and into adulthood, suggesting sex-specific vulnerability to the long-term programming effects of maternal GDM.

### Hyperglycemia directly impairs hippocampal neurogenesis in vitro

**Fig. 7:**
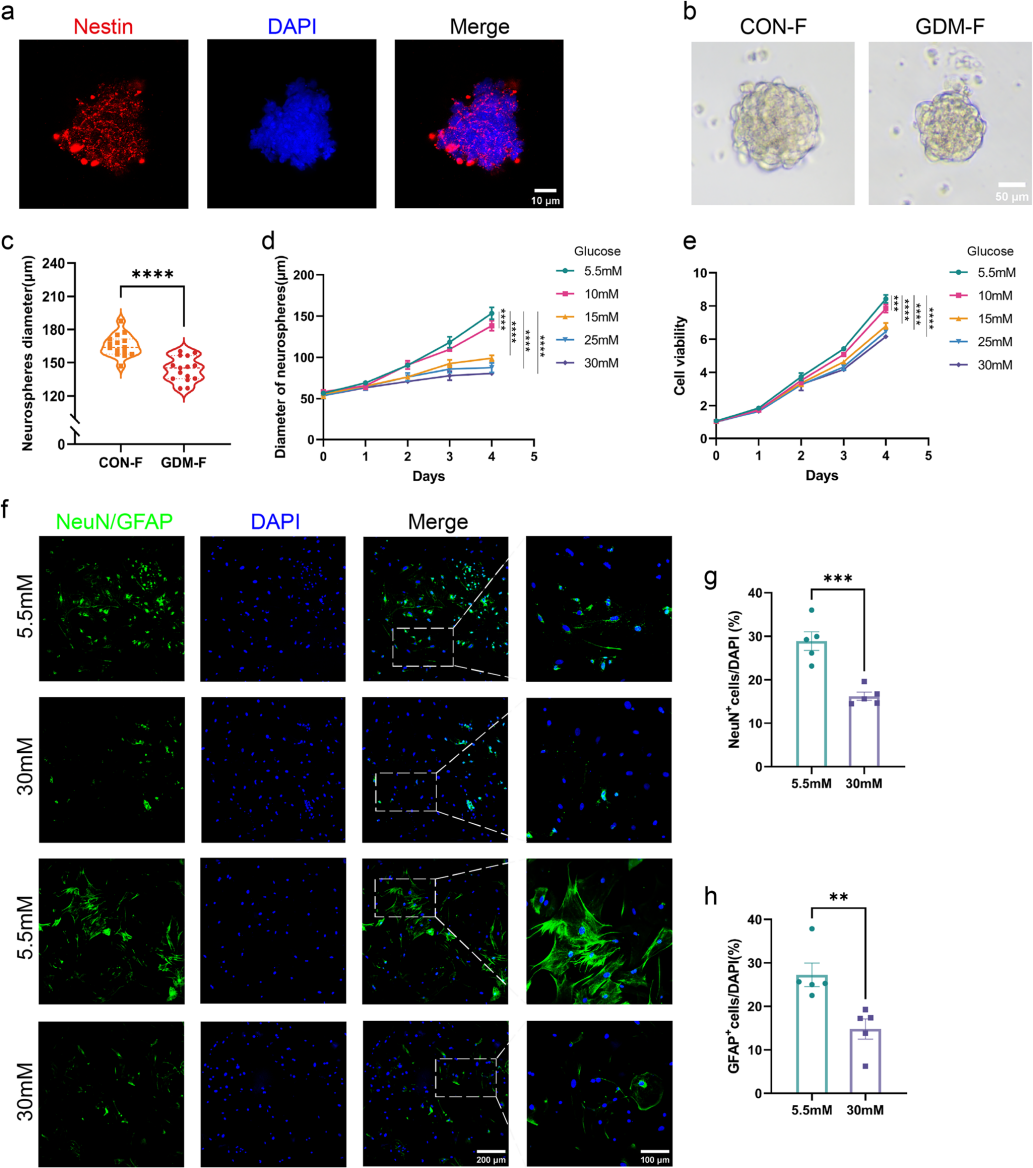
Hyperglycemia directly impairs the proliferation and differentiation of primary hippocampal NSCs. **a** Characterization of primary NSCs by immunofluorescence labeled with DAPI (blue) and Nestin (red). **b** Representative image of neurospheres. **c** Quantification of neurospheres diameter (*n* = 15). **d** Comparison of primary NSC proliferation under a glucose gradient (*n* = 5). **e** CCK-8 assay for primary NSC viability under glucose gradient (*n* = 3). **f** Representative images of primary NSC differentiated into neurons (NeuN^+^) and astrocytes (GFAP^+^) under normoglycemic (5.5 mM) or hyperglycemic (30 mM) conditions. **g**, **h** Quantification of the percentage of NeuN^+^ neurons (**g**) and GFAP^+^ astrocytes (**h**) (*n* = 5). All in vitro experiments were performed using primary NSCs isolated from the hippocampus of female E12.5 embryos. Values are presented as mean ± s.d. Statistical significance was assessed by an unpaired Student’s *t*-test. For longitudinal data, a two-way repeated measures ANOVA was used, followed by Tukey’s post hoc test to assess differences at each time point. *P < 0.05, **P < 0.01, ***P < 0.001, ****P < 0.0001.

To further validate the impact of intrauterine hyperglycemia on hippocampal neurogenesis, we isolated hippocampal NSCs from E12.5 embryos of GDM and control female mice for in vitro experiments (Fig. 7a). Primary NSC cultures from GDM female offspring formed smaller neurospheres compared with controls, suggesting altered proliferative dynamics (Fig. 7b, c). Glucose concentration-dependent assays revealed that elevated glucose levels progressively suppressed NSC proliferation and viability, with reductions in both parameters as glucose concentrations increased (Fig. 7d, e). Differentiation capacity was assessed under normoglycemic (5.5 mM) and hyperglycemic (30 mM) conditions. High glucose exposure significantly impaired differentiation into both neurons and astrocytes (Fig. 7f–h). These findings demonstrate that hyperglycemia directly disrupts hippocampal neurogenesis, recapitulating the neurogenic deficits observed in GDM female offspring.

### Metabolomic analysis reveals persistent myo-inositol depletion in GDM female offspring hippocampus

**Fig. 8:**
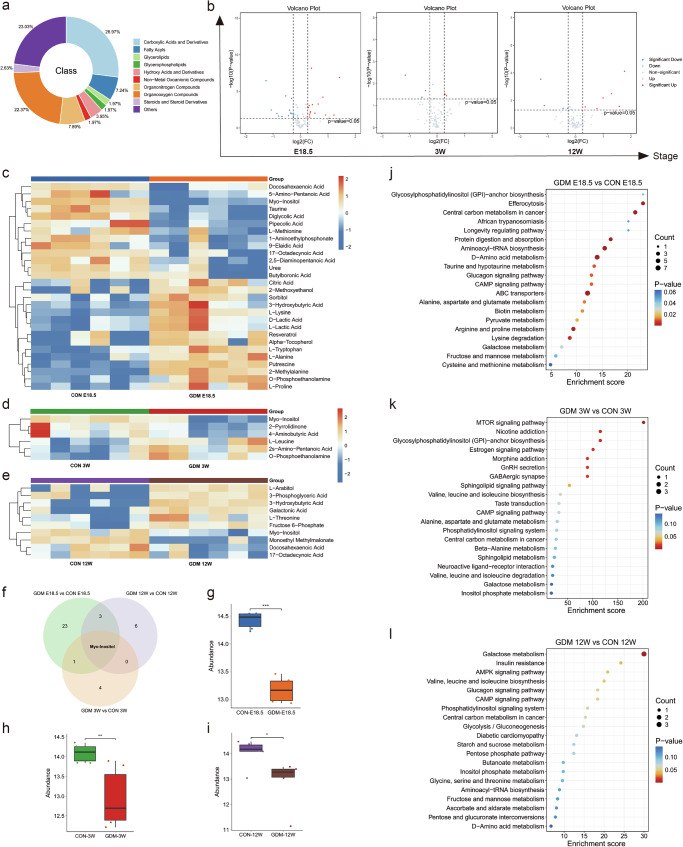
Nontargeted metabolomic analysis in the hippocampus of GDM female offspring. **a** Pie chart classifying all identified metabolites. **b** Volcano plots illustrating differentially abundant metabolites between GDM and control groups at each time point. **c**–**e** Heat maps showing the relative abundance of differential metabolites at E18.5 (**c**), 3 weeks (**d**) and 12 weeks (**e**). **f** A Venn diagram showing the overlap of differential metabolites across the three stages. **g**–**i** Box plots displaying the significant and persistent downregulation of myo-inositol in the GDM group at E18.5 (**g**), 3 weeks (**h**) and 12 weeks (**i**). **j**–**l** Bubble plots of KEGG pathway enrichment analysis at E18.5 (**j**), 3 weeks (**k**) and 12 weeks (**l**). Values are presented as mean ± s.d. (*n* = 6 per group). Statistical significance was assessed by an unpaired Student’s *t*-test. *P < 0.05, **P < 0.01, ***P < 0.001.

To elucidate the developmental effects of GDM on hippocampal neurogenesis, we performed GC–MS on hippocampal tissues from female offspring at E18.5, 3 weeks and 12 weeks. A total of 152 metabolites were identified, spanning diverse classes, including carboxylic acids and derivatives (26.97%), organooxygen compounds (22.37%) and fatty acyls (7.24%) (Fig. 8a). Differential metabolites were identified using the criteria of *P* value <0.05 and FC ≥1.2 or ≤1/1.2 (Fig. 8b). At E18.5, 28 differential metabolites were identified (15 upregulated, 13 downregulated) (Fig. 8c); at 3 weeks, 6 differential metabolites were identified (3 upregulated, 3 downregulated) (Fig. 8d); and at 12 weeks, 10 differential metabolites were identified (6 upregulated, 4 downregulated) (Fig. 8e). Cross-stage analysis via Venn diagrams revealed that myo-inositol was the only common differential metabolite, with its abundance consistently downregulated across all time points (Fig. 8f–i). Kyoto Encyclopedia of Genes and Genomes (KEGG) pathway enrichment analysis further revealed significant enrichment in pathways related to inositol metabolism (glycosylphosphatidylinositol (GPI)-anchor biosynthesis, phosphatidylinositol signaling system), neurotransmitter metabolism (alanine, aspartate and glutamate metabolism; valine, leucine and isoleucine biosynthesis) and carbohydrate metabolism (glucagon signaling pathway, galactose metabolism) across all stages (Fig. 8j–l). These findings implicate myo-inositol depletion and dysregulated inositol signaling as central drivers of the persistent neurogenic deficits observed in GDM female offspring.

### GDM-induced oxidative stress contributes to neurogenic impairments in hippocampal NSCs

Oxidative stress is implicated in the developmental programming effects of GDM on offspring. To investigate this, we assessed oxidative stress in hippocampal NSCs of female offspring. Immunofluorescence staining with anti-TOMM20 confirmed reduced mitochondrial density in the GDM hippocampus (Supplementary Fig. 4a,b). In vitro validation using primary hippocampal NSCs demonstrated that high glucose (30 mM) exposure disrupted mitochondrial dynamics. Confocal imaging revealed fragmented and aggregated mitochondrial networks in the high glucose group, compared with the filamentous networks in controls (Supplementary Fig. 4c). Functional assays further showed elevated ROS levels and diminished mitochondrial membrane potential under hyperglycemic conditions (Supplementary Fig. 4d–f). Redox homeostasis was profoundly disrupted, with reduced GSH levels, a decreased GSH/GSSG ratio, and a decreased NAD^+^/NADH ratio in the high glucose group (Supplementary Fig. 4g,h). These results establish that maternal GDM induces persistent oxidative stress in hippocampal NSCs of female offspring, characterized by mitochondrial dysfunction and redox imbalance, probably contributing to the neurogenic deficits observed.

## Discussion

Depression and anxiety represent leading neuropsychiatric burdens in adolescents, yet the mechanistic links between GDM and these disorders remain elusive^30,31^. Our study demonstrates that maternal GDM induces sex-specific behavioral outcomes: female offspring develop depressive-like behaviors without anxiety-related phenotypes, whereas males exhibit metabolic dysfunction devoid of behavioral alterations. Crucially, we implicate disrupted hippocampal neurogenesis as a female-specific pathway underlying these behavioral deficits. Developmental trajectory analysis revealed that neurogenic suppression initiates prenatally in both sexes but progresses to sustained impairment only in females—a decline paralleled by myo-inositol depletion and mitochondrial oxidative stress.

The metabolic consequences of GDM are well-established^1–4^. In this study, we combined a pregestational HFD and low-dose STZ to establish a GDM mouse model. This approach recapitulates the pathophysiology of human GDM by inducing both hyperglycemia and insulin resistance primarily in mid-to-late gestation, while avoiding potential confounders such as maternal obesity seen in high-sugar and HFD induction models, or the severe β-cell necrosis and embryotoxicity associated with high-dose STZ induction models^32^. Using this model, our study revealed that GDM affected the growth trajectory of offspring but did not substantially affect their overall developmental growth. Interestingly, male offspring displayed substantial metabolic dysfunction, characterized by impaired glucose tolerance and insulin resistance, while female offspring did not exhibit similar metabolic dysfunction. This finding is consistent with preclinical evidence from rodent models showing male-biased metabolic disturbances following adverse in utero exposures^33–35^. Notably, our sex-specific metabolic phenotype is also in line with clinical observations across different life stages. In a clinical birth cohort, male infants born to GDM mothers exhibit elevated insulin, C-peptide and leptin levels in umbilical cord blood at birth, suggesting that metabolic dysregulation may begin in utero^36^. This alteration appears to persist into childhood. In a clinical follow-up study, 9.5-year-old male offspring of GDM pregnancies show higher fasting insulin and HOMA-IR, key markers of insulin resistance^37^. Our findings of impaired glucose metabolism at weaning and in adulthood further suggest that this male-specific vulnerability is established early and persists throughout adult life. The underlying mechanisms driving this developmental programming probably involve complex and sustained interactions among hormonal, epigenetic and placental factors initiated in utero^37,38^.

Emerging evidence underscores the neurodevelopmental risks associated with GDM, yet the specific vulnerabilities appear to be strongly modulated by offspring sex^6,7,10^. However, reports on these sex-specific outcomes are inconsistent. Several studies suggest that male offspring are more susceptible to certain neurodevelopmental disorders. For example, epidemiological data have linked maternal GDM to a higher risk of ASD and ADHD in male offspring^39,40^. By contrast, our study provides compelling evidence for a distinct female-specific vulnerability, characterized by persistent hippocampal neurogenesis deficits and a subsequent depressive-like phenotype. This finding is consistent with clinical observations suggesting that females may have a higher susceptibility to mood disorders such as depression and anxiety following adverse prenatal exposures^11^. These disparate findings suggest that the impact of GDM on the developing brain is multifaceted.

The precise mechanisms by which maternal hyperglycemia impacts fetal brain development and leads to adverse behavioral outcomes remain unclear. Current theories suggest that epigenetic modifications, neuroinflammation and immunity, structural brain changes, lipid metabolism alterations and oxidative stress may all play critical roles in mediating the effects of GDM on offspring neurodevelopment^6^. Our study did not observe major structural changes in the hippocampus, possibly because such changes are more prominent in severe cases of depression^41^. However, we observed substantial impairments in synaptic plasticity in the hippocampus of female offspring exposed to GDM, characterized by a reduction in dendritic length and complexity. These alterations may disrupt the establishment of functional neural circuits, which is consistent with findings from Valle Bautista et al.^42^. Furthermore, BDNF, a key neurotrophic factor involved in neuronal differentiation and plasticity, has been shown to be essential for brain functions^6,43^. A reduction in BDNF expression in the hippocampus has been linked to cognitive deficits in offspring^44,45^. Interestingly, BDNF is also considered a mediator of glucose utilization and energy metabolism, and it has cytoprotective roles in pancreatic β-cells^46^. These findings suggest that BDNF may serve as a bridge connecting GDM exposure with neurodevelopmental impairments in offspring. However, further research is needed to explore the precise relationship between GDM, BDNF and offspring neurodevelopmental outcomes.

NSCs in the hippocampal subgranular zone possess the ability to self-renew and produce new neurons throughout life, a process that is widely considered essential for cognitive and emotional regulation^16,17,47^. Our findings indicate that GDM disrupts hippocampal neurogenesis in a sex-specific manner, impairing both NSC proliferation and differentiation. While both male and female offspring exhibit early neurogenic impairments during the embryonic period, a distinct divergence emerges postnatally: female offspring show sustained neurogenesis deficits, which correlate with their depressive-like behaviors in adulthood. By contrast, the neurogenic deficits in male offspring appear to be transient and do not persist into adulthood, consistent with their lack of behavioral alterations. These results suggest that sustained disruption of neurogenesis is a key factor driving the onset of depression in female offspring exposed to GDM. In addition, our observations imply that male offspring may possess a potential neuroprotective mechanism. Despite enduring long-term metabolic disturbances, male offspring seem to compensate metabolically, which may reduce neural damage in the hippocampus in the context of hyperglycemia. However, this compensatory mechanism requires further investigation to fully understand its effects on long-term neurodevelopment and overall health.

Furthermore, our hippocampal metabolomics highlighted disruptions in pathways critical for neurogenesis, particularly those related to myo-inositol signaling. Myo-inositol, a cyclic sugar alcohol abundant in brain tissue, regulates neuronal signaling, osmoregulation and membrane metabolism^48,49^. The persistent downregulation of myo-inositol in female offspring is probably a direct consequence of the intrauterine hyperglycemic environment. Under hyperglycemic conditions, excess glucose is shunted into the polyol pathway, where the rate-limiting enzyme, aldose reductase, consumes NADPH to convert glucose to sorbitol^50^. This increased flux through the polyol pathway can deplete the cellular pool of NADPH, a critical cofactor required for the de novo synthesis of myo-inositol from glucose by myo-inositol-1-phosphate synthase (INO1)^51^. The resulting myo-inositol depletion perturbed GPI-anchor biosynthesis and the phosphatidylinositol signaling system in female offspring, both of which are essential for maintaining normal neurogenesis. Intriguingly, myo-inositol itself is involved in glucose metabolism and insulin signaling, and its supplementation during pregnancy has been shown to prevent GDM and improve offspring metabolic outcomes^52,53^. Postnatal myo-inositol administration has also been reported to increase BDNF expression and enhance metabolic health in offspring^54,55^. Given that myo-inositol in human milk may promote neuronal connectivity, the potential of maternal myo-inositol supplementation to prevent the GDM-induced neurodevelopmental impairments observed in our study represents a promising and clinically relevant avenue for future investigation^56^.

Oxidative stress is another critical mechanism by which GDM induces neurogenic deficits in offspring. It has been well-established that hyperglycemic environments promote the generation of oxidative free radicals, leading to neuronal dysfunction and damage^57–59^. In our study, female offspring exposed to GDM exhibited increased oxidative stress and mitochondrial dysfunction, which probably contributed to the disruption of hippocampal neurogenesis.

While our findings provide novel insights into the sex-specific neurodevelopmental consequences of GDM, several limitations warrant consideration. First, while our GDM model recapitulates key aspects of human GDM pathophysiology, it also presents a challenge for direct comparison and integration with findings from other common GDM models. Second, our neurobiological analysis was focused on the hippocampus. Given that other brain regions, such as the prefrontal cortex and amygdala, are also critically involved in mood regulation, future studies should investigate whether they are similarly affected. Furthermore, our behavioral assessment was limited to anxiety- and depression-like phenotypes, and future work should expand this to include other domains reportedly affected by maternal GDM, such as social and attentional behaviors relevant to ASD and ADHD. Third, our in-depth mechanistic studies on myo-inositol metabolism and oxidative stress were confined to female offspring. The biological basis for the apparent neurogenic resilience in male offspring, despite their metabolic compromise, remains an important question for future investigation. Finally, although we identified myo-inositol depletion as a key pathological event, myo-inositol supplementation was not tested in this study. Ongoing work in our group is investigating the mechanistic role of myo-inositol metabolism in hippocampal neurogenesis and the translational implications of these pathways in GDM-exposed offspring.

In conclusion, our study reveals that maternal GDM induces sustained hippocampal neurogenesis deficits selectively in female offspring, a process driven by persistent myo-inositol depletion and oxidative stress that culminates in long-term depressive-like behaviors. These findings not only elucidate a novel mechanism for the sex-specific neurodevelopmental vulnerabilities following GDM exposure but also provide a framework for targeting metabolic-neural crosstalk. This approach may hold therapeutic potential for preventing or treating neuropsychiatric disorders linked to adverse in utero metabolic environments.

## Supplementary information

Supplementary Information

## Data Availability

The data presented in this study are available on request from the corresponding author.
