# Supplementary material for: Sex-specific effects of maternal gestational diabetes mellitus on offspring neurodevelopment: persistent hippocampal neurogenesis deficits in female but not male offspring
Source: Exp Mol Med. 2026 Jun 4;58(6):1854–69. doi: 10.1038/s12276-026-01741-z (PMC13324582; doi:10.1038/s12276-026-01741-z)
Supplement: Supplementary file 1 — Supplementary Information [file 12276_2026_1741_MOESM1_ESM.pdf]

Supplementary Information

Sex-specific Effects of Maternal Gestational Diabetes Mellitus on Offspring Neurodevelopment: Persistent Hippocampal Neurogenesis Deficits in Female but not Male Offspring

Xiafei Wu[1], Huisheng Ge[1], Jie Fang[1], Jie He, Hongbing Xu, Yangyu Zhao, Philip N Baker, Xinyang Yu[*], Yubin Ding[*], and Hongbo Qi[*]

This file includes:

Supplementary Table 1

Supplementary Fig. 1 to Fig. 4

Supplementary Table 1 Primer sequence

Gene	Forward	Reverse
Htr1a	TCCTAGAACACGCAGCTTCTTAG	GCCAAAGACCGAGCCAATAATTT
Htr2a	GCGGGAAACATACTGGTCATCAT	GGAGAAGAGCACATCCAGGTAAA
Htr3a	GTATGCCATCCTCAACGTGTACT	CGATGATGCACGTACACATAAGG
Drd1	CTGTGAAGTTTGGTGTGCCG	CACGGCTGTTCAGAAGACTCATA
Drd2	TCTCCAGGCACCAGAAGAAATTT	CTGAGTACGGTGAAGTGTCCTTTA
Drd5	TTGCTTCTGGGCAATCCTGT	AGGTTGAGCAGGACATACGC
Grin1	AGTTTGACCCAGGAACCAAGAAT	GTCAGCCGTTTCAGTCCTTATGA
Gria1	GTTGGCTGTGTATGAGGAGATGA	GACTCGTTCTTGCCGTTGATTAG
Bdnf	CCGGTATCCAAAGGCCAACT	AGGGCCCGAACATACGATTG
Gapdh	ACTCTTCCACCTTCGATGCC	TGGGATAGGGCCTCTCTTGC

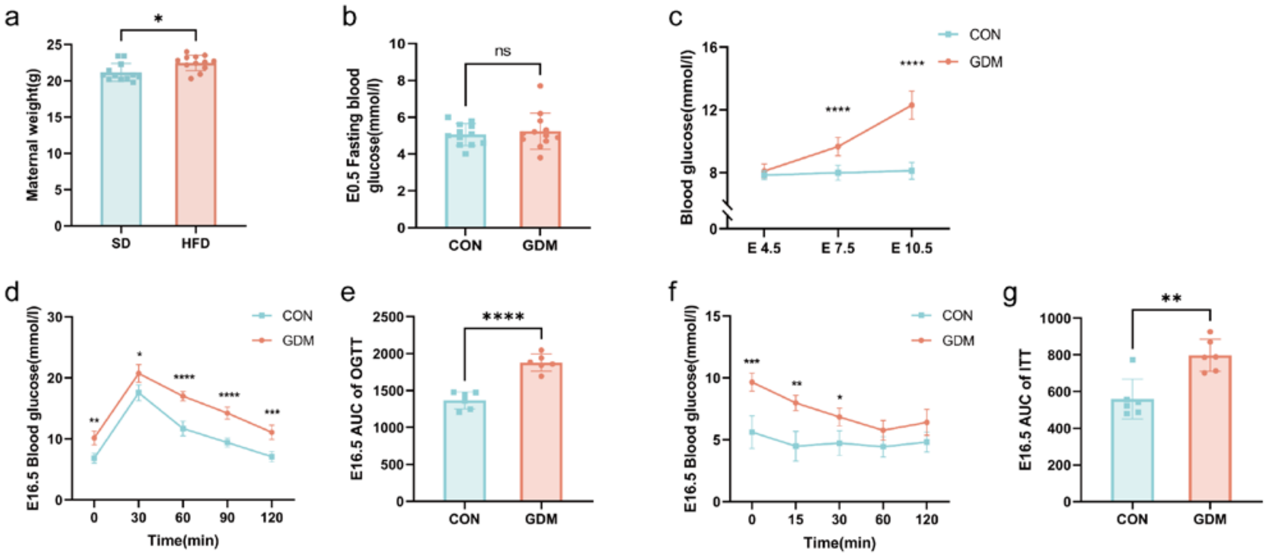

Supplementary Fig. 1: Maternal metabolic alterations induced by GDM. (a) Body weight of dams fed a high-fat diet (HFD) versus a standard diet (SD) at the end of the 4 weeks pregestational period (n = 12). (b) Fasting blood glucose levels in dams at E0.5 (n = 12). (c) Random blood glucose levels in dams during early to mid-gestation (n = 12). (d) Oral glucose tolerance test (OGTT) curves in dams during late gestation (E16.5) (n = 6). (e) Area under the curve (AUC) quantification for the OGTT (n = 6). (f) Insulin tolerance test (ITT) curves at E16.5 (n = 6). (g) AUC quantification for the ITT (n = 6). Values are presented as mean ± SD. Statistical significance was assessed by an unpaired Student's t-test. For longitudinal data, a two-way repeated measures ANOVA was used, followed by Bonferroni's post hoc test to assess differences at each time point. * $P < 0.05$, ** $P < 0.01$, *** $P < 0.001$, **** $P < 0.0001$.

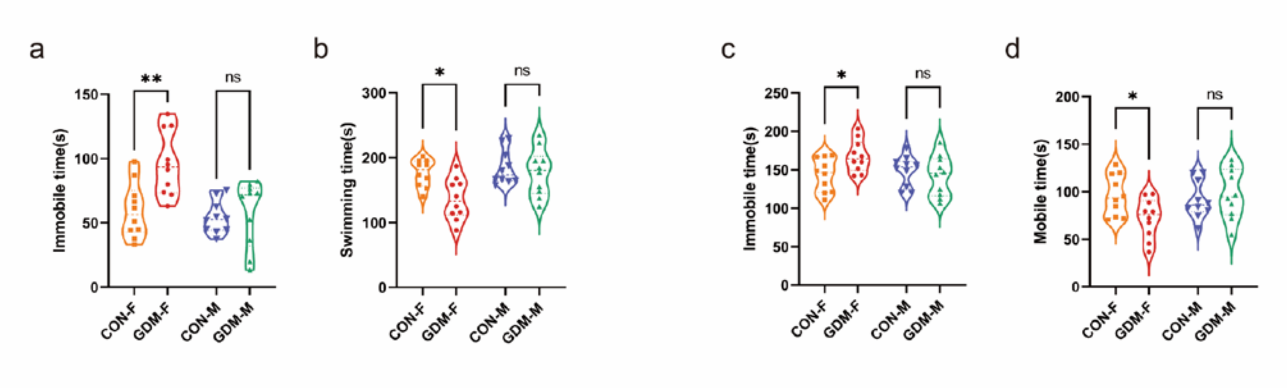

Supplementary Fig. 2: Sensitivity analysis of depressive-like behaviors using a 4-minute statistical window. To assess the robustness of our findings, data from the forced swim test (FST) and tail suspension test (TST) were re-analyzed using a 4-minute time window, consistent with standardized protocols. (a/b) Quantification of immobility time (a) and swimming time (b) in the FST (n = 10). (c/d) Quantification of immobility time (c) and mobility time (d) in the TST (n = 10). The results are consistent with the primary 5-minute analysis presented in Figure 3, confirming the depressive-like phenotype in female GDM offspring. Values are presented as mean ± SD. Statistical significance was assessed by a two-way ANOVA with Tukey's post hoc test. * $P < 0.05$, ** $P < 0.01$, indicating a significant difference between GDM and control groups of the same sex.

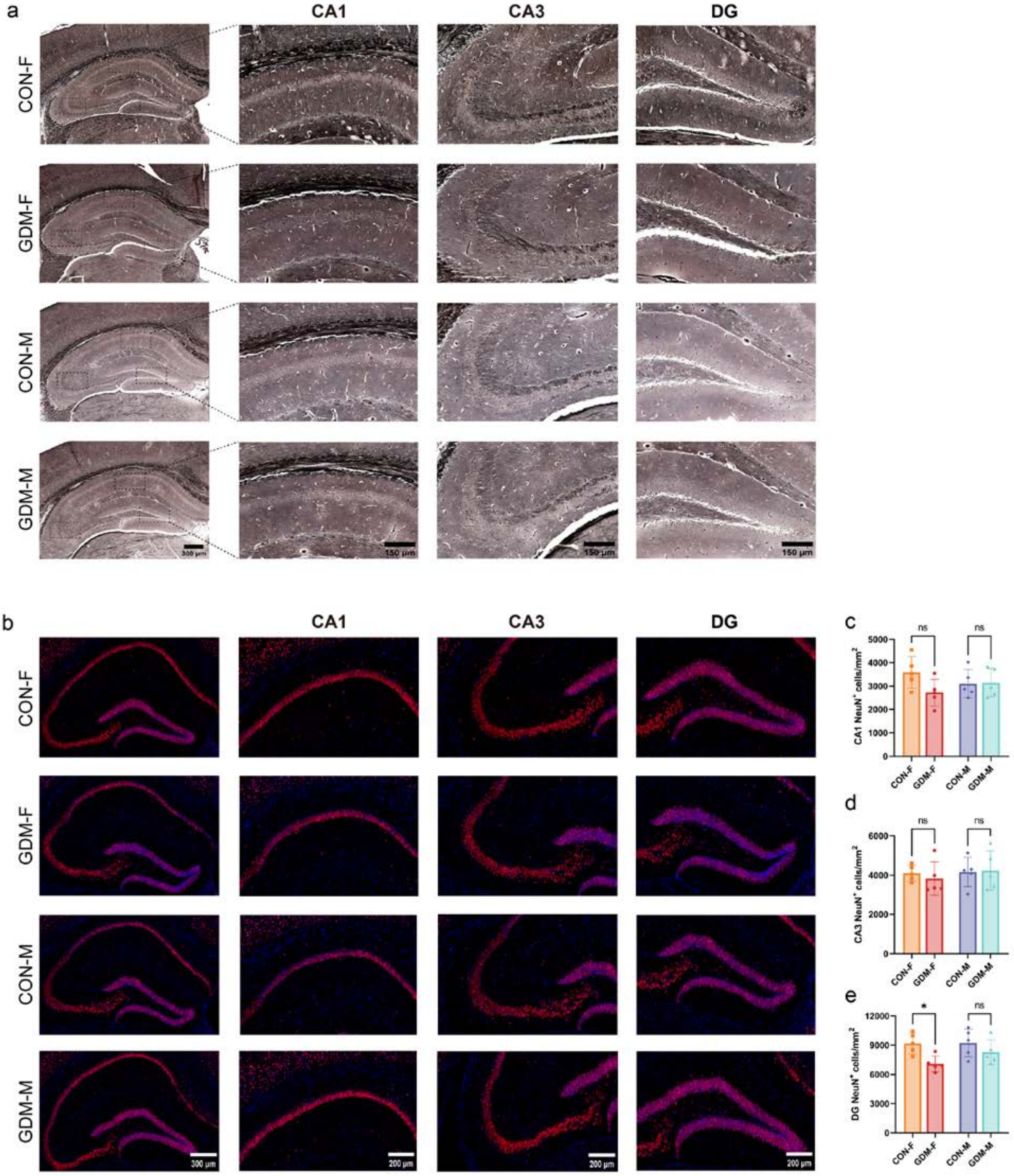

Supplementary Fig. 3: Assessment of neurodegeneration and mature neuron populations in the hippocampus of adult offspring. (a) Representative images of Bielschowsky's silver staining in the hippocampus of adult offspring (12W). (b) Representative immunofluorescence images for the mature

neuron marker NeuN (red) in the hippocampus of adult offspring (12W). (c-e) Quantification of NeuN-positive cell density in the CA1 (c), CA3 (d), and DG (e) regions (n = 5). Values are presented as mean ± SD. Statistical significance was assessed using a two-way ANOVA followed by Tukey's post hoc test. * $P < 0.05$.

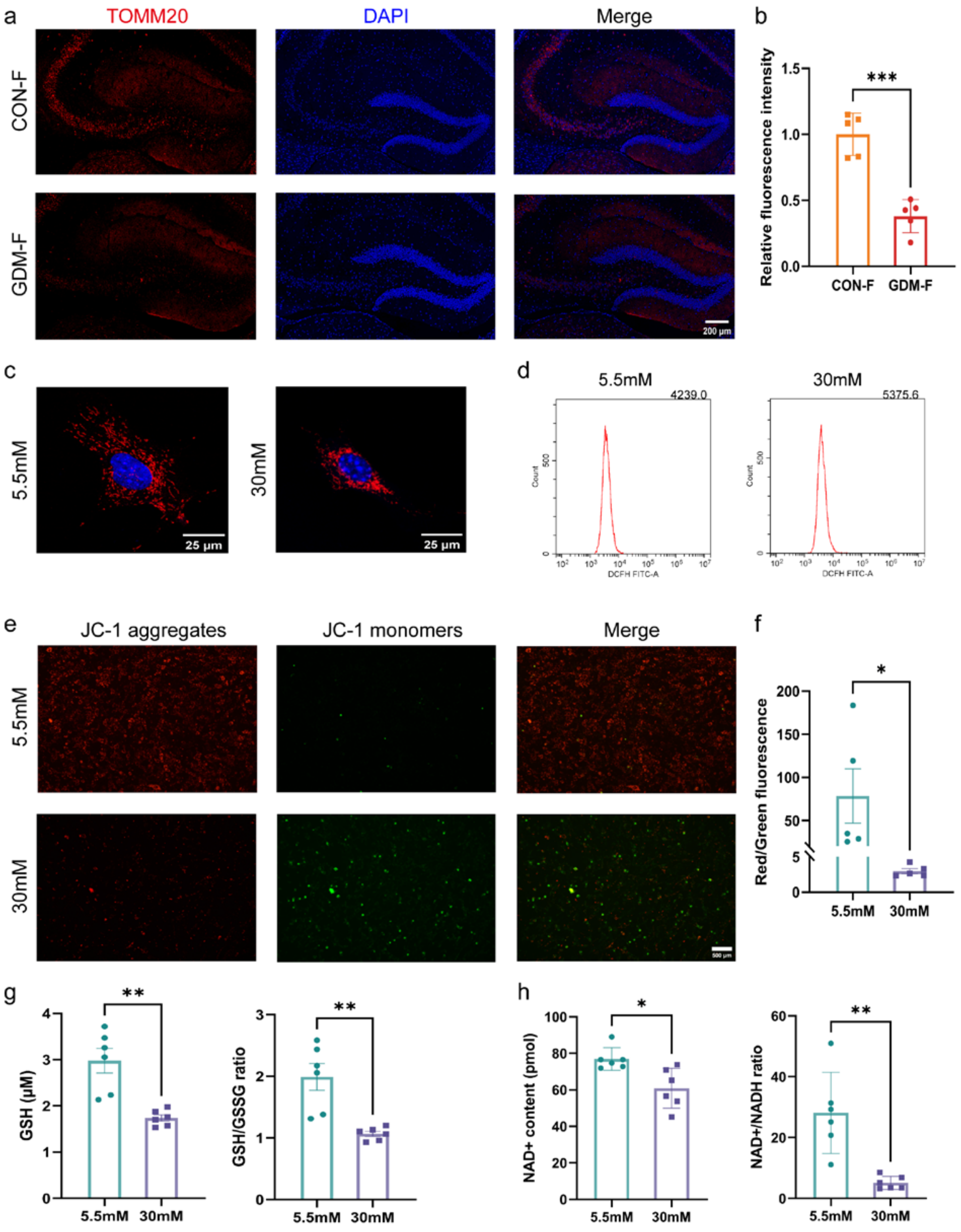

Supplementary Fig. 4: GDM induces oxidative stress in hippocampal NSCs of female offspring.

(a) Representative immunofluorescence images of TOMM20-labeled (red) mitochondria in the

hippocampus of adult female offspring (12W). (b) Quantification of TOMM20 fluorescence intensity (n = 5). (c) Representative confocal images of TOMM20-stained mitochondria in primary NSCs cultured under normoglycemic (5.5 mM) or hyperglycemic (30 mM) conditions. (d) Flow cytometry analysis of ROS levels in primary NSCs. (e, f) Representative images of JC-1 staining (e) and quantification of the red/green fluorescence ratio (f) in primary NSCs (n = 5). (g) Glutathione (GSH) levels and the GSH/GSSG ratio in primary NSCs (n = 6). (h) NAD^+ levels and the NAD^+/NADH ratio in primary NSCs (n = 6). Values are presented as mean ± SD. Statistical significance was assessed by an unpaired Student's t-test. * $P < 0.05$, ** $P < 0.01$, *** $P < 0.001$, **** $P < 0.0001$.
